# Inequalities in birth weight and maternal education: a time-series study from 1996 to 2013 in Brazil

**DOI:** 10.1038/s41598-020-65445-8

**Published:** 2020-05-26

**Authors:** Sonia Silvestrin, Vânia Naomi Hirakata, Clécio Homrich da Silva, Marcelo Zubaran Goldani

**Affiliations:** 1 Technical Area for Child and Adolescent Health, Porto Alegre Municipal Health Department, Porto Alegre, RS 90040-971 Brazil; 2 Research and Graduate Studies Group, Hospital de Clínicas de Porto Alegre, Porto Alegre, RS 90035-007 Brazil; 3 Department of Pediatrics, Federal University of Rio Grande do Sul, Porto Alegre, RS 90035-003 Brazil

**Keywords:** Neonatology, Epidemiology, Risk factors

## Abstract

Maternal education represents one of the most important social determinants of inequality in birth weight (BW) in developing countries. The present study sought to investigate secular trends in health inequality considering the difference in mean BW between extremes of maternal educational attainment in Brazil. Using a time-series design, data from 6,452,551 live births which occurred in all Brazilian state capitals from 1996 to 2013 were obtained from the Information System on Live Births. Secular trends of the difference in mean birth weight between low (<8 years of schooling) and high (≥12 years of schooling) educational attainment were analyzed. The main finding was that differences in mean birth weight between the two extremes of maternal educational attainment decreased over time. There was a significant decrease in mean BW in neonates born to mothers with higher educational attainment, and a slight increase in those born to mothers with lower educational attainment. One of the key factors involved in decreasing inequality was an increase in the number of antenatal visits. In view of these results, we conclude, that despite a slight increase of mean birth weight among mothers with low education, the reduction of inequality in pregnancy outcomes over time in Brazil is attributable to a worsening scenario for mothers who are better off rather than to improvements for the most vulnerable group of mothers.

## Introduction

Socioeconomic status is considered an important predictor of health inequalities. Individuals in unfavorable socioeconomic conditions are more vulnerable to adverse physical and mental health outcomes compared to those who are better off^1^.

Several demographic and socioeconomic conditions influence birth outcomes. Among them, ethnicity, education, occupation, income, social class, the availability of housing, urban infrastructure, social support, and exposure to crime have all been described^2^.

The impact of social inequalities on human health is well established, and has shown increasing relevance in developing countries. Thus, in these countries, it is important to encourage social and economic development policies which, together with public health programs, may favorably influence perinatal outcomes, particularly birth weight^3,4^. At the individual level, access to health care, medical practices, behavioral factors, and stress are also related to birth weight^5,6^.

Maternal education is a strong determinant of birth weight acting through a number of mediators such as adequate access to information, health care and nutrition^7^. In Brazil, we have shown in a previous study, despite significant improvements in maternal education, the rate of LBW remained stable around 8.5% of all life births during the last 15 years^8^. In Brazil, this situation regarding LBW is considered an epidemiological paradox and, according to some researchers, it can be explained by several factors, among them, the underreporting in the registry of low birth weight in the poorest and most vulnerable regions and, at the same time, the intense use of health care technologies such as cesarean sections and assisted reproduction techniques in other wealthier regions with more favorable socioeconomic conditions in the country^9^.

Birth weight has impacts that persist throughout the life cycle, and its determinants are many^6,10^. Biological, demographic, socioeconomic, and environmental factors during pregnancy have a significant influence on birth weight^10–14^. Among these, maternal social integration is considered one of the most relevant. Mothers exposed to stress are more likely to engage in high-risk behaviors such as smoking, alcohol intake, and psychoactive drug use, and are more likely to be overweight or obese—all factors which have been associated with lower birth weight^15^. Maternal education is also among the most relevant social determinants of child health; mothers with low educational attainment are at higher risk of delivering low birthweight infants^7,8,16,17^.

Average birth weight has increased in many countries over the last three decades, due to improved access to antenatal care and population-wide improvements in socioeconomic condition^4,18^. However, some recent studies have found that mean birth weight has declined in some developed countries, although specific causes have not been identified^10,19^. Therefore, mean birth weight has presented different secular trends among different countries, under the influence of several determinants.

Brazil has one of the highest rates of low birth weight among developing countries, at approximately 8,0% and remaining stable over the last 10 years^8,20^. This rate is strongly influenced by sociodemographic factors and by access to adequate health care, and thus reflects high inequality among the different social classes and geographic regions of the country. However, the assessment of rates of low birth weight alone is not able to show the small impacts of the various individual social determinants of birth weight over time^9,21^.

Therefore, the objective of this study is to evaluate the influence of inequality between extremes of maternal education on birth weight, considering the trend of absolute and relative differences in mean birth weight over time among mothers with higher and lower educational attainment in an 18-year time series (1996–2013) in Brazil.

## Methods

### Data source and study population

This time-series study included all live births which occurred in 26 Brazilian state capitals and the Federal District from 1996 to 2013. Data were obtained from the Information System on Live Births (*Sistema de Informação sobre Nascidos Vivos*, SINASC), which covers 96% of all live births in Brazil^22^. This open-access database is available through the Ministry of Health website (http://datasus.saude.gov.br) or directly on the Health Information page of the Unified Health System Department of Informatics – DATASUS (http://datasus.saude.gov.br/informacoes-de-saude/tabnet).

Multiple births were excluded, as were newborns weighing <500 g or >8,000 g. These exclusions represented 6.02% of all live births during the period of interest (Table 1). The decision to use only information about births occurring in Brazilian capitals was based on the recognition that, in these cities, epidemiological surveillance services are more consolidated and have a more robust structure, which ensure greater completeness and reliability of data^23^.

**Table 1:** Number of exclusions according to the pre-established criteria.

Variable	n (%)
Multiple deliveries	253,937 (1.98)
Missing Information on birth weight	52,461 (0.40)
Births outside state capitals	486,754 (3.64)
Total	793,152 (6.02)

Overall, there were 6,452,551 live births in Brazilian state capitals and the Federal district during the study period, representing 11.92% of all live births in the country. Assessment for completeness showed a missing data rate <0.5%^22,23^.

### Outcome, variable, and covariables of interest

Trends in mean birth weight (in grams) over time, presented as a continuous variable, were the outcome of interest. The investigated variable, maternal educational attainment, was dichotomized into two extremes: low and high (<8 and ≥12 years of formal schooling, respectively). Thus, extreme levels of maternal education were used because we understand that these categories are more subject to the potential impact of access to health services and technologies during antenatal and perinatal care on birth weight^24^.

The following covariables were considered: maternal age (10–17, 18–34, ≥35 years), number of previous live births (0 or ≥1), number of antenatal visits (none, <6, or ≥7 visits), gestational age (˂37 or ≥37 weeks), and mode of delivery (vaginal or cesarean). State capitals were grouped by the five geographic regions of Brazil^25^.

### Statistical analysis

Birth weight means were calculated yearly for each Brazilian capital. The absolute and relative differences in mean birth weight between the two extremes of maternal education were calculated to assess the inequality between them.

The annual means of birth weight differences between the two extremes of maternal education was estimated using a linear mixed model^26^ for the years 1996 to 2003 and 2004 to 2013. We split in two periods of time after analysis using Joinpoint Regression Program Software Environment (version 4.3.1). The variables year and geographical region of birth were entered into the initial (standard) model (Model a). The covariables maternal age (Model b), number of children (Model c), number of antenatal visits (Model d), gestational age (Model e), and mode of delivery (Model f) were sequentially included.

Statistical analysis was performed in PASW Statistics, Version 18.0 (SPSS Inc., Chicago, IL), with a 5% significance level and 95% confidence intervals (CIs).

### Ethical aspects

The study protocol was approved by the Research Ethics Committee of Hospital de Clínicas de Porto Alegre (Brazil) with opinion number 16–0338. The study was carried out in accordance with national and international guidelines.

## Results

During the study period, there was a significant reduction in adolescent pregnancies. There was also an increase in cesarean sections among mothers with low educational attainment, which, by the end of the period, was equivalent to approximately half of the percentage observed among mothers with high educational attainment. The descriptive variables used in the study, including maternal socioeconomic conditions, number of previous live births, number of antenatal visits, percentage of pregnancies in adolescence, cesarean sections, and preterm deliveries, are shown in Table 2.

**Table 2:** Percent distribution of descriptive variables (maternal socioeconomic conditions: number of previous live births, number of prenatal visits, teenage pregnancy, cesarean section, preterm newborns) in Brazilian state capitals, 1996–2013.

Year of birth	Maternal education	Number of primiparous mothers (%)	<6 antenatal visits (%)	Cesarean section (%)	Preterm birth (<37w) (%)	Teenage pregnancy (10–17 y.o.) (%)
1996	<8 years	*	57.8	28.0	5.6	10.1
	≥12 years	*	9.8	78.6	4.7	
1997	<8 years	25.6	58.6	29.4	5.2	10.4
	≥12 years	40.5	9.2	78.2	4.9	
1998	<8 years	29.3	61.9	28.1	6.8	10.3
	≥12 years	48.5	7.4	78.9	5.0	
1999	<8 years	30.3	61.1	29.9	6.1	10.0
	≥12 years	46.5	19.7	68.2	5.3	
2000	<8 years	28.2	58.5	29.6	6.0	9.8
	≥12 years	46.5	20.3	67.0	6.1	
2001	<8 years	27.2	58.0	29.9	6.6	9.6
	≥12 years	47.2	18.0	68.5	6.0	
2002	<8 years	24.3	56.8	29.7	6.7	9.4
	≥12 years	44.3	17.1	69.2	6.4	
2003	<8 years	25.6	55.6	30.5	6.9	9.2
	≥12 years	48.1	15.1	70.8	6.9	
2004	<8 years	26.4	55.3	32.3	6.9	8.8
	≥12 years	50.3	15.5	70.5	6.9	
2005	<8 years	26.7	55.9	33.2	6.9	8.7
	≥12 years	50	15.3	72.5	6.9	
2006	<8 years	28.4	55.0	33.5	6.8	8.5
	≥12 years	53.7	15.4	73.5	6.9	
2007	<8 years	29.8	55.7	34.5	6.5	8.2
	≥12 years	54	14.7	74.8	7.0	
2008	<8 years	31.1	55.0	35.4	7.0	8.1
	≥12 years	54.6	14.7	75.9	7.2	
2009	<8 years	32.1	53.9	35.9	7.6	8.1
	≥12 years	56.1	14.5	77.6	7.1	
2010	<8 years	32.2	54.6	37.4	7.2	7.9
	≥12 years	56.2	14.0	79.2	7.1	
2011	<8 years	28.9	55.5	37.8	10.9	8.1
	≥12 years	57.5	14.1	82.3	9.1	
2012	<8 years	28.6	54.6	37.4	13.0	8.0
	≥12 years	28.9	14.7	83.5	10.4	
2013	<8 years	28.8	47.7	38.1	12.2	7.9
	≥12 years	58.2	14.7	83.0	9.6	

*No data available.

(Source: MS/SVS/DASIS - Information System on Live Births).

The findings point out a gradual decrease in the frequency of women with low educational attainment, from 55.4% in 1996 to 18.5% in 2013. Accordingly, there was an increase in the frequency of women with high educational attainment, from 7.2% in 1996 to 24.4% in 2013 (Table 3).

**Table 3:** Secular trend of maternal educational attainment in Brazilian state capitals, 1996–2013 (Source: MS/SVS/DASIS - Information System on Live Births).

Year of birth	Maternal educational attainment	
	<8 years, n (%)	≥12 years, n (%)
1996	293,937 (55.4)	38,359 (7.2)
1997	362,848 (52.9)	54,279 (7.9)
1998	353,879 (52.1)	52,622 (7.8)
1999	258,147 (48.4)	71,601 (13.4)
2000	346,399 (49.6)	117,359 (16.8)
2001	318,397 (46.9)	116,384 (17.1)
2002	290,745 (43.9)	117,172 (17.7)
2003	270,388 (40.7)	119,775 (18.0)
2004	248,155 (37.4)	128,457 (19.4)
2005	235,518 (35.9)	127,694 (19.5)
2006	213,062 (32.6)	136,235 (20.8)
2007	194,767 (30.2)	140,328 (21.8)
2008	181,329 (27.7)	150,389 (22.9)
2009	171,939 (26.2)	152,963 (23.4)
2010	159,087 (24.4)	158,829 (24.3)
2011	148,315 (22.3)	152,506 (22.9)
2012	132,038 (19.9)	153,959 (23.2)
2013	122,796 (18.5)	161,894 (24.4)
Overall	4,301,746 (36.7)	2,150,805 (18.4)

The absolute difference in mean birth weight among infants born to mothers with high vs. low educational attainment was 90.22 g in 1996, gradually decreasing to 5.05 g in 2011—the smallest difference for the whole period of interest. The relative difference also decreased, from 2.87% in 1996 to 0.41% in 2013 (Table 4).

**Table 4:** Secular trends of mean birth weight and its relative difference according to maternal educational attainment, 1996–2013 (Source: MS/SVS/DASIS - Information System on Live Births).

Year of birth	Birth weight (g)	Birth weight (g), stratified by maternal educational attainment		Relative difference (%)
		<8 years	≥12 years	
1996	3188 (528.4)	3160 (532.3)	3251 (504.6)	2.87
1997	3179 (532.1)	3162 (537.4)	3217 (506.4)	1.73
1998	3173 (530.1)	3155 (536.7)	3208 (504.0)	1.67
1999	3183 (529.4)	3169 (534.7)	3209 (507.6)	1.26
2000	3186 (530.2)	3173 (534.2)	3213 (517.1)	1.26
2001	3176 (530.6)	3166 (537.6)	3202 (507.9)	1.13
2002	3172 (531.5)	3161 (539.9)	3194 (510.9)	1.04
2003	3161 (530.8)	3149 (536.9)	3183 (512.9)	1.07
2004	3167 (534.5)	3155 (545.5)	3188 (512.7)	1.04
2005	3177 (535.7)	3167 (547.5)	3189 (511.5)	0.69
2006	3181 (540.6)	3173 (554.5)	3187 (514.3)	0.44
2007	3180 (537.7)	3173 (551.9)	3181 (513.6)	0.25
2008	3184 (540.4)	3173 (556.9)	3188 (514.8)	0.47
2009	3181 (541.6)	3170 (559.4)	3182 (515.0)	0.37
2010	3179 (537.7)	3165 (557.3)	3180 (511.1)	0.47
2011	3180 (537.8)	3166 (560.0)	3171 (509.0)	0.15
2012	3187 (540.5)	3170 (562.2)	3178 (507.5)	0.25
2013	3187 (538.7)	3166 (563.4)	3179 (505.9)	0.41
**Overall**	3179 (535.0)	3164 (544.1)	3189 (511.3)	0.78

Data given as mean (SD) unless otherwise noted.

**Figure 1:**
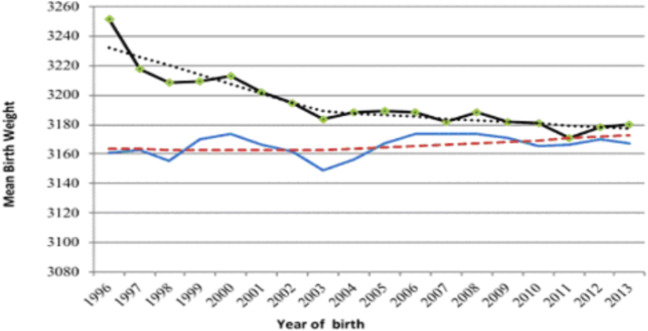
Secular trends and annual mean birth weight of neonates born to mothers with high and low educational attainment, 1996–2013. The Fig. 1 shows the secular trends and annual mean birthweight of neonates born to mothers with high and low educational attainment in Brazilian state capitals from 1996 to 2013. In this figure, the curves of the mean weight of newborns born to mothers with high (“Mean_high”) and low (“Mean_low”) education are presented, as well as the secular trends of average weight of newborns born to mothers with discharge (“Jp high”) and low (“Jp low”) education, assessed through Joinpoint Regression. Each of these curves has different outlines and colors marked each year. It is possible to observe that the mean birth weight among neonates born to mothers with high educational attainment declined steadily throughout the study period, while remaining stable among infants born to mothers with low schooling, with only a slight increase from 2005 onward.

Mean birth weight among neonates born to mothers with high educational attainment declined steadily throughout the study period, while remaining stable among infants born to mothers with low schooling, with only a slight increase from 2005 onward (Fig. 1).

**Figure 2:**
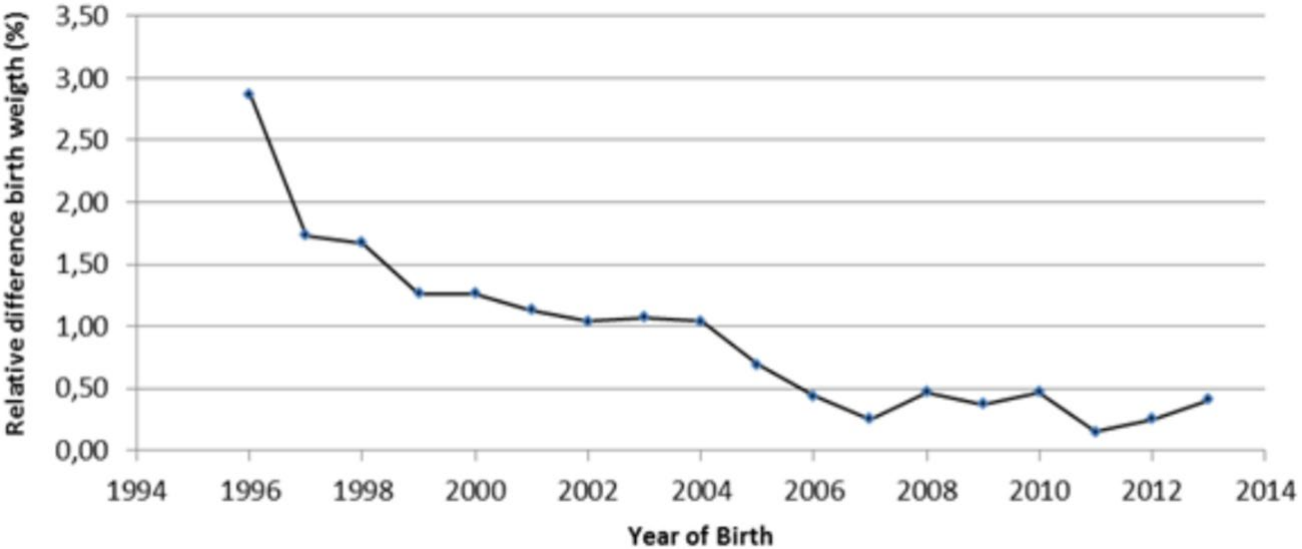
Secular trend of relative difference in mean birth weight among neonates born to mothers with high and low educational attainment, 1996–2013. The Fig. 2 shows the secular trend of relative difference in mean birth weight among neonates born to mothers with high and low educational attainment in Brazilian capitals from 1996 to 2013. This figure shows a single curve constructed from the difference between the mean birth weight of newborn children of mothers with higher education and the mean birth weight of newborn children of mothers with less education, year by year. This relative difference in mean birth weight between the two extremes of maternal education showed a downward trend over the study period.

The relative difference in mean birth weight between the two extremes of maternal education showed a downward trend over the study period (Fig. 2).

Table 5 presents the means of birth weight differences of neonates born to mothers with high vs. low educational attainment, adjusted for covariables, in a sequential linear mixed model in the two different periods of analysis.

**Table 5:** Mixed linear models with estimates of the annual difference in birth weight (g) among singleton neonates born alive to mothers living in Brazilian capitals with low versus high (reference) educational attainment, adjusted for covariates, in 1996–2003 and 2004–2013.

Model	Low vs High educational attainment					
	1996–2003			2004–2013		
	Difference in birth weight (g)	95%CI	*P*	Difference in birth weight (g)	95%CI	*P*
a	11.42	(9.04; 13.81)	<0.001	1.47	(0.43; 2.52)	0.005
b	10.37	(7.79; 2.95)	<0.001	2.25	(1.10; 3.41)	<0.001
c	10.76	(7.37; 4.14)	<0.001	1.63	(0.19; 3.08)	0.02
d	14.63	(11.67; 17.59)	<0.001	3.60	(2.12; 5.09)	<0.001
e	10.63	(8.43; 12.84)	<0.001	1.03	(0.04; 2.01)	0.04
f	10.80	(6.32; 15.28)	<0.001	1.03	(−0.77; 2.84)	0.26

^a^Adjusted for geographic region (place of birth): North (reference), Northeast, Central West, Southeast, and South.

^b^Adjusted for maternal age: 18–34 years (reference).

^c^Adjusted for number of previous live births: multiparous (reference). ^d^Adjusted for number of antenatal visits: ≥ 7 (reference).

^e^Adjusted for length of gestation: ≥37 weeks (reference).

^f^Adjusted for type of delivery: cesarean section (reference).

In both periods, the number of antenatal visits (**Model d**) reduced the inequality between birth weight means in the two extremes of maternal education. Maternal age (**Model b**) had a negative effect, mainly in the second period (2004–2013), increasing the inequality in means of birth weight between the two extremes of maternal education. The covariables number of children, gestational age, and mode of delivery did not show any effect on the differences in birth weight means between the two groups of educational attainment during the study period.

## Discussion

The results of this study showed a significant decline in mean of birth weight among infants born to mothers with a higher education level, leading to a significant reduction of general mean of birth weight during the time series. In terms of inequality in means of birth weight according to maternal education, we present a scenario compatible with the hypothesis of similarities in the inequality proposed for us previously, in which intense use of technologies and care coexisting with lack of access to them can lead a similar outcome^9,27,28^. Besides, the general increase in maternal education could contributed to amplify the severity of inequality between the two extremes of social, even though we noticed a narrowing trend of the outcome. In this case, the less educated mothers represent the most vulnerable social group in the country leading to an scarce access to health care and educational policies during the study period.

In addition, several maternal and child health indicators—including number of antenatal visits, multiparity, and the teenage pregnancy rate—showed overall improvement during the study period^29^. In contrast, there was an increase in primiparous women aged 35 or older, which has been associated with the increase in cesarean sections and premature births in Brazil^8,30,31^.

Other factors could be playing a significant role in this scenario, such as improvements in income distribution, with a general reduction in poverty leading to less maternal malnutrition, better quality of health care, and increased access to health care, especially for underprivileged social groups^31^. Besides, social programs, such as conditional cash programs, showed significant impact on infant health leading to reduction of infant mortality in less privileged communities^32^.

However, these positive factors did not produce a detectable effect on birth weight for women in unfavorable socioeconomic conditions, possibly indicating that this group has experienced extreme social exclusion and marginalization. Thus, considering this scenario, the stability of mean birth weight in the less educated group can be considered a net positive.

The effect of the number of antenatal visits on birth weight could be explained in two different directions according to educational level. For more educated mothers, antenatal care can be associated with intensive use of new technologies, such as *in vitro* fertilization, cesarean section, and ultrasound, leading to possible reductions in mean birth weight even without an increase in preterm birth rates. In mothers with lower schooling, expanded access to antenatal care can lead to better care of basic health needs, contributing to a reduction in acute illness and increased vaccination and nutritional follow-up rates^29–31^.

Antenatal care has long been considered an important factor related to birth weight. Inadequate numbers of antenatal visits are associated with low birth weight^33–35^, while increased access to antenatal care is associated with improved birth weight and other favorable pregnancy outcomes^27^. Mothers who attended fewer than 6 visits had a relative risk for low birth weight of 2.47 compared to those who attended 6 or more visits, even after adjustments for income and maternal education^36^. Six or more antenatal visits were associated with better outcomes for pregnant women with hypertensive disorders, while fewer visits or no antenatal care whatsoever were related to stillbirth and perinatal death, as well as higher risk of urinary tract infections, chorioamnionitis, and streptococcal vaginitis^37,38^.

The steady increase in rates of cesarean section presented influence in reduction in the difference in mean birth weight in both study periods, but more so in the second period proportionally. These increases occurred in women at both extremes of maternal education, but in those with lower educational attainment. The greater proportional increase in CS rates in this less privileged group of women could represent significant improvement in access to adequate health care, instead of excess of intervention, amplifying fetal survival, initially for heavy newborns. Brazil has one the highest rates of cesarean section in the world (52%)^39^, which has been associated with high rates of preterm birth^35^. This practice may also be associated with changes in the concept of fetal viability and interventions to prevent fetal deaths^10,40^.

Preterm births also have played a role in the birth weight trends observed herein, especially in the group of mothers with higher educational attainment. A study that evaluated preterm births between 2000 and 2010 in the most developed areas of Brazil suggested that successful interventions increased the rate of preterm live births by 45.2%^40^. Likewise, changes in the profile of pregnant women in the country, with an increase in the number of mothers aged 35 or older, have increased the risk of pre-existing health conditions and complications during pregnancy, such as hypertensive disorders^40,41^. This factor could be related to the decrease in mean birth weight observed, and has been previously described as the “low birth weight paradox”, whereby better socioeconomic development is associated with a higher rate of low birth weight^28^.

Limitations of this study include the lack of information about preexisting maternal clinical background, obstetric diseases, and intercurrent events during pregnancy. Data on pre-gestational body mass index, smoking, alcohol intake, psychoactive drug use, and maternal nutrition were not available. Inaccurate recording of gestational age in weeks and the high rate of incompleteness of the variable race/skin color made the inclusion of these variables in the study impossible.

On the other hand, the national scope of this study, the number of live births included in the analyses (more than 6 million) and its representativeness within the Brazilian population, the 18-year period of analysis (from 1996 to 2013), and the acknowledgment of maternal obstetric characteristics associated with birth weight are among the strengths of this study.

These results reflect the demographic, epidemiological, and perinatal transitions in the country over the last two decades, which have had an impact on mean birth weight. Similar trends have been reported in other countries, such as the United States and China, with decreases in birth weight in the last year^10,12,20^. Social advances and improvements in maternal education and maternal and child care have failed to exert a positive impact on birth weight, probably due to increased medical and technological interventions at all maternal education levels. Besides, the limited change in mean birth weight observed among infants born to women in unfavorable socioeconomic conditions may suggest that advances have not reached the most vulnerable strata of society.

In conclusion, despite a slight increase of mean birth weight among mothers with low education, this study indicates that the reduction of social inequalities in birth weight in Brazil came at the expense of worse outcomes in the better-off group, as opposed to improvements in mothers with low educational attainment. These findings suggest that the current epidemiological profile of birth outcomes in Brazil is characterized by the presence of “equality in inequality”, whereby similar outcomes occur as a result of differential effects of interventions and different social scenarios, leading to a merely apparent reduction of social inequality between extreme social strata.

Finally, the results found allow us to affirm that the reduction of inequality in pregnancy outcomes over time in Brazil is mostly attributable to a worsening scenario for mothers who are better off rather than to improvements for the most vulnerable group of mothers.
